# Circulating Exosomal miR-1-3p from Rats with Myocardial Infarction Plays a Protective Effect on Contrast-Induced Nephropathy via Targeting ATG13 and activating the AKT Signaling Pathway

**DOI:** 10.7150/ijbs.55887

**Published:** 2021-03-02

**Authors:** Pengcheng Zhao, Yeqian Zhu, Ling Sun, Wenwu Zhu, Yao Lu, Jian Zhang, Yangming Mao, Qiushi Chen, Fengxiang Zhang

**Affiliations:** 1 Section of Pacing and Electrophysiology, Division of Cardiology, the First Affiliated Hospital with Nanjing Medical University, Nanjing, China.; 2 Department of Cardiology, the Affiliated Changzhou No. 2 People's Hospital of Nanjing Medical University, Changzhou, China.

**Keywords:** Contrast-induced nephropathy, Contrast medium, Iodixanol, Exosome, MiR-1-3p, ATG13, AKT signaling pathway.

## Abstract

**Rationale:** With the widespread development of the interventional technique for cardiovascular diseases and the widespread use of contrast medium (CM), the incidence of contrast-induced nephropathy (CIN) has been increasing, which is associated with poor prognosis for cardiovascular diseases. This study aims to explore the effect of circulating exosomal microRNA from patients with myocardial infarction (MI) on CIN and related molecular mechanism. **Methods:** A rat MI model was established by ligating the left anterior descending coronary artery. Circulating exosomes were isolated from control (Exo-NC) and MI rats (Exo-MI) using a commercial kit. The *in vivo* and *in vitro* models of CIN were created using iodixanol. Reverse transcription quantitative PCR (RT-qPCR) was utilized to detect the expression of miR-1-3p. Western blot (WB) was used to detect the expression of exosomal surface markers, and apoptosis-related and autophagy-related proteins. The apoptosis rate was examined by terminal deoxynucleotidyl transferase (TdT)-mediated dUTP nick end labeling (TUNEL) staining and flow cytometry (FC). Transmission electron microscopy (TEM) was utilized to observe the exosomes and autophagosomes. Rat kidney injury was assessed by hematoxylin and eosin (H&E) staining and kidney injury molecule-1 (KIM-1) immunohistochemical staining. Renal function of rats was assessed by detecting the levels of blood urea nitrogen (BUN) and serum creatinine (Cr). The dual luciferase reporter assay was performed to identify the target gene of miR-1-3p. **Results:** The treatment of CM induced NRK-52E cell damage, which manifested as enhanced cell autophagy and enhanced apoptosis. The Exo-MI treatment significantly inhibited the CM-induced autophagy and apoptosis of NRK-52E cells. Furthermore, the Exo-MI treatment increased the Bcl-2 expression, but decreased the Bax expression and the ratio of LC3II/LC3I. Furthermore, the results of the TUNEL staining and FC showed that Exo-MI can reduce apoptotic rate. Through TEM, it was found that Exo-MI reduced the number of autophagosomes in NRK-52E cells. The rescue experiments revealed that the function of Exo-MI is to inhibit the CM-induced autophagy and apoptosis of NRK-52E cells, which can be inhibited by the miR-1-3p inhibitor. Furthermore, it was found that the overexpression of miR-1-3p can also inhibit the CM-induced autophagy and apoptosis of NRK-52E cells. Through dual luciferase reporter assay, ATG13 was found to be the target of miR-1-3p. In addition, the overexpression of miR-1-3p significantly reversed the CM-induced decrease in phosphorylation level of AKT. Furthermore, ATG13 silencing can also inhibit the CM-induced autophagy and apoptosis of NRK-52E cells. *In vivo*, Exo-MI significantly alleviated the renal injury, reduced the renal fibrosis, and improved the renal function of CIN rats. **Conclusion:** The circulating exosomal miR-1-3p after MI inhibited the CM-induced apoptosis and autophagy of renal tubular epithelial cells, and improved the renal function of rats by targeting ATG13 and activating the AKT signaling pathway.

## Introduction

Acute myocardial infarction (AMI) is one of the most common emergencies in the cardiovascular system. With the development of interventional therapy, the prognosis of patients with AMI has been greatly improved 1, 2. Meanwhile, the application of contrast medium (CM) during interventional thearpy may lead to the increased risk of contrast induced nephropathy (CIN), which is assosiated with adverse short- and long- term outcomes of patients.

CIN is defined as acute impairment of renal function within 2-7 days after intravenous CM 3, 4. With the wide application of percutaneous coronary intervention (PCI), CIN has become one of the leading cause of iatrogenic renal failure 5, which is associated with permanent renal impairment and higher risk of in-hospital and 1-year mortality 6, 7. However, treatment of CIN is mainly supportive or renal replacement therapy. It is urgent to find a new therapeutic targets for CIN.

Exosomes are extracellular vesicles (30-150 nm) released by all cell types and were originally thought to be cellular garbage 8. Over the past few decades, some studies have confirmed that exosomes are rich in specific cargoes (proteins, lipids, nucleic acids, etc.) 9, 10. These cargoes are then transferred to recipient cells and regulate cellular functions.

Myocardial microRNAs are released into the circulation after AMI. However, how they impact remote organs is still clear. In the present study, we show that circulating miR-1-3p is carried in exosomes and mediate functional crosstalk between the ischemic heart and the kidney. We found that following cardiac injury, exosomal miR-1-3p is rapidly released in a remarkable quantity to the kidney, in which suppress ATG13 expression and mediate renal apoptosis and autophagy. Thus, our studies reveal a novel pathway of a response to cardiac ischemic injury, which may be leveraged for cell based cardiovascular repair.

## Materials and Methods

### Rat MI and CIN model establishing

All animal procedures were approved by the Committee on the Use of Live Animals in Teaching and Research at the Nanjing Medical University, and were performed according to the guidelines of the National Institutes of Health Guidelines for the Care and Use of Laboratory Animals.

Sprague-Dawley (SD) (body weight 200-240 g, male) rats were obtained from Beijing Vital River Laboratory Animal Technology Co., Ltd. (Beijing, China). All rats were kept at a constant temperature (21℃-24℃), and were given free access to water and standard chow diet.

The establishment of the rat MI model was performed, as previously described 11. Rats were anesthetized with 10% chloral hydrate and fixed on hardwood boards, followed by skin preparation on the left chest. The tracheal cannula was inserted through the mouth of rats and attached to a small animal ventilator. The left anterior descending coronary artery was ligated at the level between the pulmonary artery cone and the left atrial appendage. It can be observed that the redness turned white from the ligation site to the apex of the heart. Then, we closed the thoracic cavity, disinfected the incision with iodophor, and injected 100,000 units of penicillin to prevent infection. After 24 hours, the blood of rats was collected to extract the exosomes.

The creation of the rat CIN model performed, as previously described 12. Rats were anesthetized with 10% chloral hydrate. We cut the skin from the corner of the rib ridge on the back of the rat, bluntly separated the muscle and fat tissue, exposed and ligated the left renal pedicle, and sutured the muscle and skin layer by layer. At one week after the operation, iodixanol (10 mL/kg) (HENGRUI MEDICINE, Shanghai, China) was injected via the tail vein. After 24 hours, the blood and kidney tissues of rats were collected. CIN rats were injected with circulating exosomes from MI rats or control rats via the tail vein.

### Exosomes isolation

The collected rat blood was first centrifuged (3,000 rpm × 10 minutes) to obtain the plasma. The Exosome Isolation Reagent (RiboBio, Guangzhou, China) was used to extract the circulating exosomes, according to manufacturer's instructions. First, the plasma samples were centrifuged (2,000 g × 10 minutes) to remove the cell debris. Then, the supernatant was transferred to a new tube with the Ribo exosome isolation reagent at a volume ratio of 3:1. After the mixture was uniformly mixed, this was incubated at 4°C overnight. Finally, the mixture was centrifuged (15,000 g × 2 minutes) to obtain the exosomes.

### Cell culture and transfection

NRK-52E cells were cultured in Dulbecco's Modified Eagle's Medium/F12 (DMEM/F12, Gibco, Rockville, MD, USA) containing 10% fetal bovine serum (FBS, Biological Industries, Beijing, China) and 1% penicillin/streptomycin (Gibco, Rockville, MD, USA), and placed in an incubator containing 5% CO2 at 37℃. When the confluence of cells in the culture dish reached 80%, the cells were seeded in a 6-well plate, 24-well plate, or 96-well plate, according to the purpose of the experiment. Iodixanol (HENGRUI MEDICINE, Shanghai, China) was used to establish the *in vitro* model of CIN. For the functional studies of Exo-MI, Exo-MI or Exo-NC were co-cultured with NRK-52E cells. For the functional studies of miR-1-3p, miR-1-3p mimics or mimics negative control (mimics-NC) (RiboBio, Guangzhou, China) were transfected into NRK-52E cells using the riboFECTTM CP Transfection Kit (RiboBio, Guangzhou, China) according to the protocols. For the functional studies of ATG13, plasmids that contain ATG13 small hairpin RNA (shR-ATG13) or negative control small hairpin RNA (shR-NC) (GeneChem, Shanghai, China) were transfected into NRK-52E cells to knockdown ATG13.

### Transmission electron microscope (TEM)

The extracted exosomes were suspended in phosphate buffered saline (PBS), and 20 μL of the suspension was dropped on a copper-loaded mesh with a pore size of 2 nm. Then, this was allowed to stand at room temperature for 2 minutes, and the liquid was blotted from the side of the filter with filter paper. The sample was stained with 30 μL of 3% phosphotungstic acid solution for five min at room temperature. The solution was blotted dry with filter paper, air-dried at room temperature, and the morphology of the exosomes was observed under TEM.

The NRK-52E was collected and fixed with 2.5% glutaraldehyde for 2 hours. Then, the cells were fixed with 1% osmium tetroxide, and embedded in an epoxy resin. Afterwards, the sections were cut and stained with uranyl acetate and lead citrate. The autophagosomes were observed under TEM.

### Nanoparticle tracking analysis

The extracted exosomes were suspended in PBS. Nanoparticle tracking analysis (NTA) was implemented to detect the size distribution and concentration of particles using ZetaView PMX 110 (Particle Metrix, Germany).

### Exosome tracking

The red lipophilic fluorescent dye Dil (ThermoFisher, USA) was used to label the exosomes. Briefly, Dil was incubated with exosomes for 15 minutes. Then, the Exosome Isolation Reagent was added, and the mixture was centrifuged (1,500 g × 30 minutes) to remove the excessive dye. Afterwards, the Dil-labelled exosomes were co-cultured with NRK-52E cells for different time durations (0, 12, and 24 hours). Next, the cells were fixed with 4% paraformaldehyde for 20 minutes. DAPI (Beyotime, Shanghai, China) was used to stain the nucleus. The staining was observed using a Confocal Laser Scanning Microscope.

### Cell Counting Kit-8 assay

Cell Counting Kit-8 (CCK-8) assay was performed to detect the cytotoxicity of iodixanol. NRK-52E was seeded in 96-well plates, and treated with different concentrations (0, 30, 60, 90 and 120 mg/mL) of iodixanol for different periods of time (2, 4, 6, and 8 hours). Cell viability was detected using Cell Counting Kit-8 (YEASEN, Shanghai, China), according to the protocols. The absorbance at 450 nm was detected using a microplate reader.

### Western blot

NRK-52E cells were seeded in 6-well plates. The total protein of cells was extracted using a protein extraction kit (KeyGEN BioTECH, Nanjing, China). The concentration of protein was measured using a BCA protein quantitative analysis kit (ThermoFisher, USA). Then, the protein samples were mixed with loading buffer (Beyotime, Shanghai, China), and the protein was denatured by boiling.

The SDS-PAGE gel preparation kit (Beyotime, Shanghai, China) was used to prepare the gels of appropriate concentration based on the relative molecular mass of the protein. After the electrophoresis was completed, the protein was transferred to polyvinylidene fluoride (PVDF) membranes (ThermoFisher, USA). The membranes were incubated with 5% skim milk to block non-specific antigens, followed by incubation with the primary antibodies (Bax, Bcl-2, LC3, ATG13, CD63, Tsg101, AKT, p-AKT and GAPDH, all from Cell Signaling Technology, USA). Afterwards, the membranes were incubated with the secondary antibody. Finally, the protein blots were developed using the Molecular Imager ChemiDoc XRS + System.

### RNA extraction and Reverse transcription quantitative PCR

The total RNA of NRK-52E cells was extracted with TRizol Reagent (ThermoFisher, USA), and the total RNA of exosomes was extracted using the QIAzol Lysis Reagent (QIAGEN, GER).

The miRNA 1st Strand cDNA Synthesis Kit (Vazyme, Nanjing, China) was used for the miRNA cDNA synthesis, according to the protocols. The miRNA Universal SYBR qPCR Master Mix (Vazyme, Nanjing, China) was used to perform the reverse transcription quantitative PCR (RT-qPCR). U6 was used as the internal control of miR-1-3p in cells, and cel-miR-39 was used as the external control of miR-1-3p in exosomes. All primer sequences are listed in Table 1.

### Flow cytometry

NRK-52E cells were seeded in 6-well plates. After different treatments, the cell supernatant was collected, and adherent cells were collected with trypsin without EDTA (Gibco, USA). Then, the cells were washed with PBS and collected by centrifugation, and repeated for three times. Afterwards, the cells were suspended in 100 μL of Binding Buffer (KeyGEN BioTECH, Nanjing, China). The experimental groups were treated with 5 μL of Annexin V-FITC (KeyGEN BioTECH, Nanjing, China) and 5 μL of PI (KeyGEN BioTECH, Nanjing, China) in the dark. The control group included the following: a blank control group, a single stained Annexin V-FITC group, and a single stained PI group. After 15 minutes of reaction, these were analyzed by flow cytometry (FC).

### Terminal Deoxynucleotidyl Transferase Mediated dUTP Nick End Labeling Staining

The Terminal Deoxynucleotidyl Transferase (TdT)-Mediated dUTP Nick End Labeling (TUNEL) Apoptosis Detection Kit (Beyotime, Shanghai, China) was utilized to label the apoptotic NRK-52E cells and apoptotic kidney tissue. NRK-52E cells were fixed with 4% paraformaldehyde for 30 minutes, washed once with PBS, and incubated with 0.3% Triton X-100 (Beyotime, Shanghai, China) for 5 minutes. Then, the cells were washed twice with PBS, and 50 μL of TdT incubation buffer was used to incubate the cells for one hour at 37℃ in the dark. Subsequently, the cells were washed for three times with PBS. For the paraffin sections of kidney tissues, proteinase K was used to incubate the kidney tissue samples for 20 minutes. Then, 50 μL of TdT incubation buffer was utilized to incubate the samples for one hour at 37℃ in the dark after the samples were washed for three times with PBS. DAPI (Beyotime, Shanghai, China) was used to stain the nucleus. The results were observed under a fluorescent inverted microscope.

### Dual luciferase reporter assays

Luciferase reporters (RiboBio, Guangzhou, China) containing wild-type or mutant 3'UTR of ATG13 (ATG13-WT or ATG13-MUT) were constructed. HEK-293T cells were seeded into 24-well plates. When the cells grew to approximately 80% confluence, the luciferase reporters that contained ATG13-WT or ATG13-MUT, along with the miR-1-3p mimics or mimics-NC, were co-transfected into HEK293T cells using Lipofectamine® 3,000 (ThermoFisher, USA). Then, after 48 hours of transfection, the cells were collected. The Dual Luciferase Reporter Assay kit (YEASEN, Shanghai, China) was used to detect the luciferase activity, according to the instructions. Firefly luciferase activities were normalized to Renilla luciferase activity.

### Assessment of renal injury and renal function

The blood and kidneys of the rats were collected. At 24 hours after the injection of iodixanol (10 mL/kg) through the tail vein of the rats, the levels of blood urea nitrogen (BUN) and serum creatinine (Cr) were detected using the BUN content detection kit (Solarbio, Beijing, China) and Cr detection kit (Shanghai yuanye Bio-Technology, Shanghai, China), according to the instructions of the manufacturers. Hematoxylin and eosin (H&E) staining and kidney injury molecule-1 (KIM-1) immunohistochemical staining were used to assess the degree of renal injury. The kidney tissue samples were fixed with 4% paraformaldehyde. Then, these were dehydrated, seeded, dipped in wax, embedded, and sectioned to approximately 5 μm in thickness. Afterwards, the samples were stained with hematoxylin and eosin. For KIM-1 immunohistochemical staining, and the samples were deparaffinized and hydrated. Then, the samples were subjected to antigen retrieval and primary antibody incubation (KIM-1, Cell Signaling Technology, USA), followed by secondary antibody incubation. Subsequently, the samples were visualized with the DAB Substrate for the peroxidase kit (YEASEN, Shanghai, China). The results were observed under an upright microscope.

### Masson's trichrome staining

On the 7th day after the injection of iodixanol (10 mL/kg) through the tail vein of the rats, the kidneys of the rats were collected. Briefly, the kidney tissues were fixed, embedded, and sectioned into 5-µm slices. Then, the sections were stained using Weigert's iron hematoxylin solution for five minutes, and stained using Biebrich Scarlet-acid Fuchsin solution for two minutes at room temperature. Afterwards, these were counterstained with Aniline Blue for five minutes, followed by incubation in 1% acetic acid for two minutes at room temperature. The extent of the fibrosis was measured using ImageJ.

### Statistical analysis

The data were expressed as mean ± standard deviation (SD). Statistical analysis was performed using the GraphPad Prism8 software. The comparison between two groups was analyzed using student's t-test. The comparison between multiple groups was performed using one‐way analysis of variance, followed by post-hoc test. P<0.05 was considered statistically significant.

## Results

### Identification of circulating exosomes

**Figure 1:**
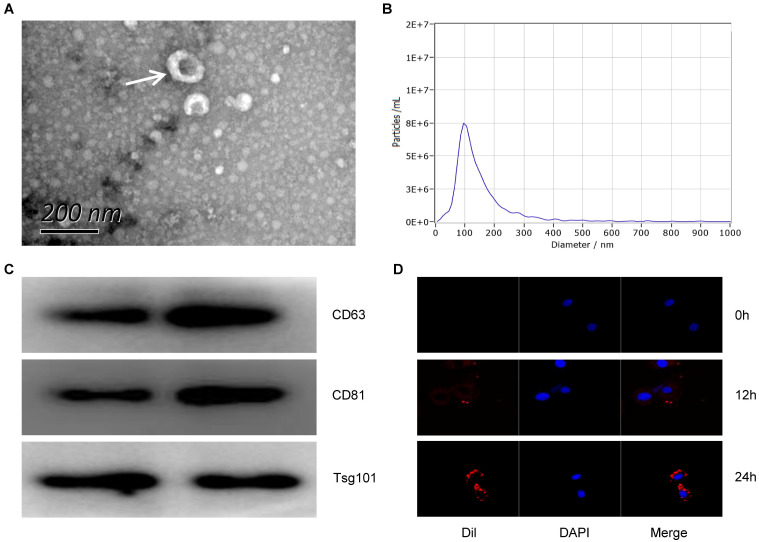
The characterization and identification of circulating exosomes. (A) The morphology of circulating plasma exosomes was observed using TEM. The white arrow indicates the exosome. Scale bar, 200 nm. (B) The particle size and particle concentration of circulating plasma exosomes were detected by NTA. (C) The expression of exosome surface markers CD63, CD81 and Tsg101 was detected by WB. (D) Dil-labeled (red) circulating plasma exosomes were taken up by NRK-52E cells. The nuclei of NRK-52E cells were stained with DAPI (blue).

In order to detect the characteristics of Exo-MI, we collected blood at 24 hours after the rat MI model establishment. Round or oval exosomes with a bilayer membrane structure were observed by using TEM (Fig. 1A). The NTA revealed that the diameter of the exosomes was approximately 100 nm (Fig. 1B). The surface markers CD63, CD81 and TSG101 of exosomes were all positive detected by WB (Fig. 1C). Figure 1D showed that the exosomes could be taken up by NRK-52E cells. These results indicate that exosomes were successfully extracted from the plasma of MI rats.

### Exo-MI inhibited the CM induced apoptosis and autophagy of NRK-52E cells

**Figure 2:**
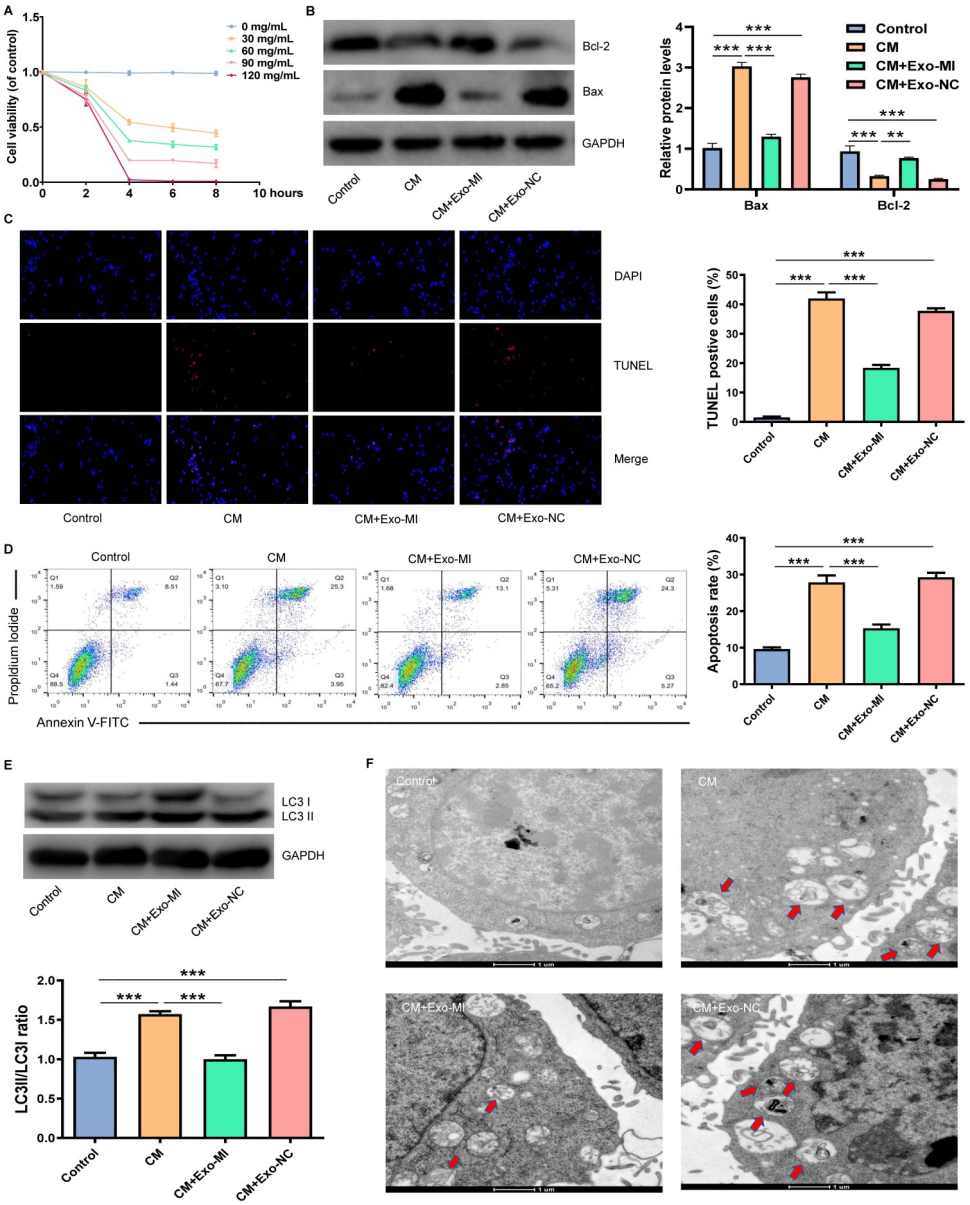
Exo-MI inhibited CM-induced apoptosis and autophagy of NRK-52E cells. (A) Different concentrations (0, 30, 60, 90 and 120 mg/mL) of CM were used to treat NRK-52E cells for different periods of time (2, 4, 6 and 8 hours), and cell viability was detected by CCK-8 assay. (B) After incubation with Exo-MI and Exo-NC with NRK-52E cells, the expression of Bcl-2 and Bax in NRK-52E cells was detected by WB. (C) The apoptosis of NRK-52E cells was detected by TUNEL staining (red). Magnification, ×200. (D) The apoptosis rate of NRK-52E cells was detected by FC. (E) The levels of autophagy-related proteins LC3I and LC3II in NRK-52E cells were detected by WB. (F) The morphology of autophagosomes was observed by TEM. The red arrow indicates the autophagosomes. Scale bar, 1 μm. The data were expressed as mean ± standard error of mean (n=3). *P<0.05, **P<0.01, ***P<0.001.

Initially, an *in vitro* model of CIN was constructed using iodixanol (30 mg/mL). Then, the Exo-MI or Exo-NC was co-cultured with NRK-52E cells. Different doses of CM were co-cultured with NRK-52E cells for different periods of time. We found that the treatment of cells with CM at a concentration of 30 mg/mL for four hours could maintain the cell viability approximately 50% (Fig. 2A). Hence, CM at a concentration of 30 mg/mL was used for the subsequent experiments. Then, the expression of apoptosis-related proteins Bax and Bcl-2 was detected by WB (Fig. 2B). NRK-52E cells were co-cultured with iodixanol resulting in a marked increase and decrease in Bax and Bcl-2 expression respectively. However, when NRK-52E cells were treated with Exo-MI, the expression of Bax and Bcl-2 expression was reversed, while Exo-NC did not have this effect. The TUNEL staining revealed that Exo-MI could protect NRK-52E cells from the apoptosis induced by CM. However, Exo-NC had no such protective effects (Fig. 2C). In addition, FC showed that Exo-MI significantly inhibit the apoptosis of NRK-52E cells induced by CM (Fig. 2D). Autophagy-related proteins LC3I and LC3II were also detected by WB (Fig. 2E). Compared with the CM group, the LC3II/LC3I ratio greatly decreased in the CM + Exo-MI group. This indicates that Exo-MI can inhibit the contrast-induced autophagy. In addition, the TEM revealed that the number of autophagosomes significantly increased in the CM group compared with the control group. However, when Exo-MI was co-cultured with NRK-52E, the number of autophagosomes markedly decreased (Fig. 2F). In summary, these results indicate that Exo-MI can inhibit the CM-induced apoptosis and autophagy of NRK-52E cells.

### The miR-1-3p increasedly expressed in Exo-MI and the miR-1-3p silencing eliminated the protective effect of Exo-MI on CIN *in vitro*

**Figure 3:**
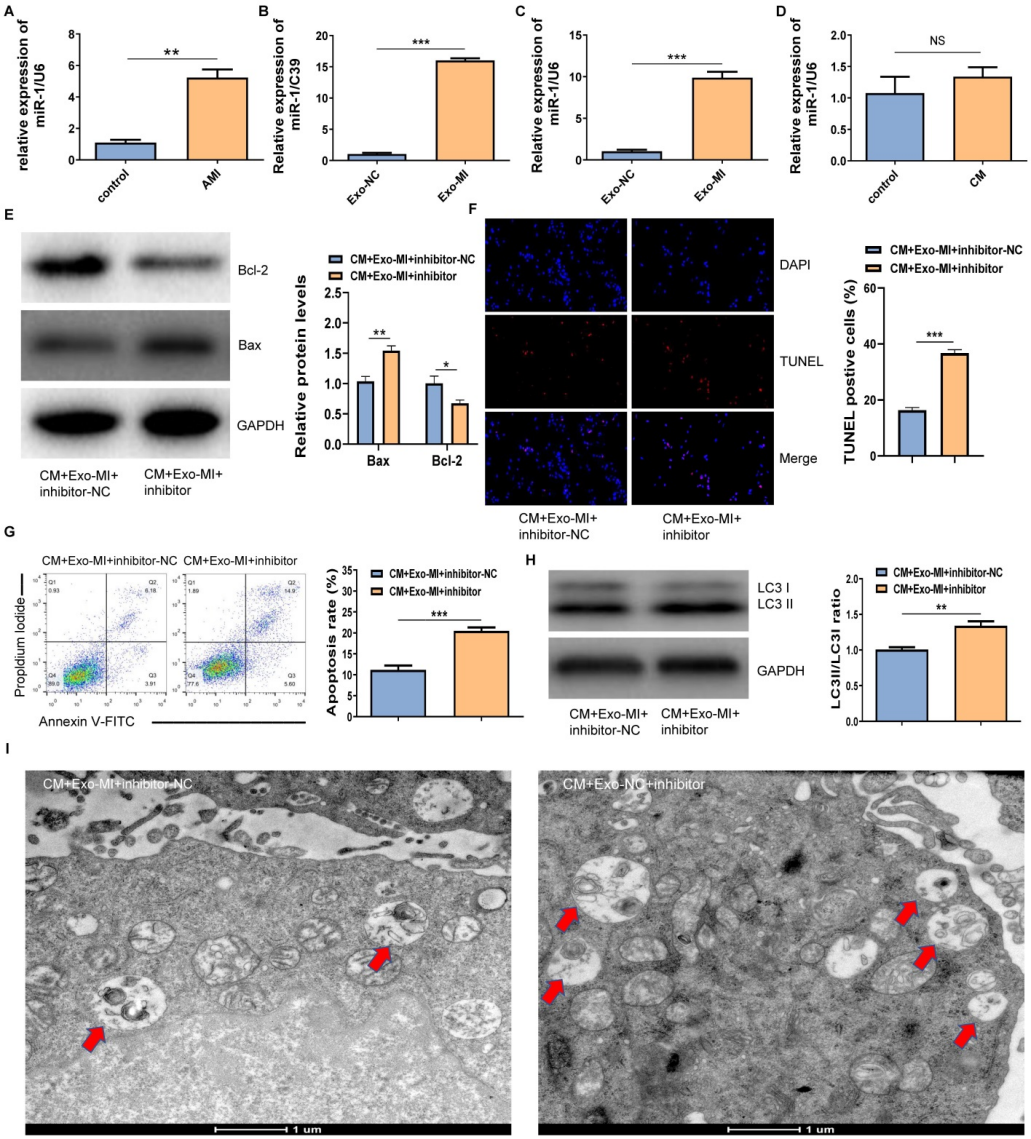
The miR-1-3p expression increased in Exo-MI rats, and the miR-1-3p silencing eliminated the protective effect of Exo-MI on CIN *in vitro*. (A) The expression of miR-1-3p in the heart tissue of rats with acute myocardial infarction (AMI) was detected by RT-qPCR. (B) The expression of miR-1-3p in Exo-MI was detected by RT-qPCR. (C) The levels of miR-1-3p in NRK-52E cells incubated with Exo-MI or Exo-NC were detected by RT-qPCR. (D) The expression of miR-1-3p in NRK-52E cells treated with CM was detected by RT-qPCR. (E) The WB analysis revealed the expression of Bcl-2 and Bax in NRK-52E cells incubated with Exo-MI and transfected with the miR-1-3p inhibitor or inhibitor-NC. (F) Apoptosis of NRK-52E cells was detected by TUNEL staining (red). Magnification, ×200. (G) The apoptosis rate of NRK-52E cells was detected by FC. (H) The levels of autophagy-related proteins LC3I and LC3II in NRK-52E cells were detected by WB. (I) The morphology of autophagosomes was observed by TEM. The red arrow indicates the autophagosomes. Scale bar, 1 μm. The data were expressed as mean± standard error of mean (n=3). *P<0.05, **P<0.01, ***P<0.001, NS represents no statistical significance.

The RT-qPCR revealed that miR-1-3p was highly expressed not only in the infarcted myocardium (Fig. 3A) but also in Exo-MI (Fig. 3B) compared with the control group. WhenNRK-52E cells co-cultured with exosomes, the expression of miR-1-3p significantly increased in NRK-52E cells (Fig. 3C). However, NRK-52E cells treated with CM did not change the level of miR-1-3p (Fig. 3D). In order to prove whether the protective effect of Exo-MI on CIN mainly depends on miR-1-3p, we conducted rescue experiments. NRK-52E cells were transfected with the miR-1-3p inhibitor or inhibitor-NC, then co-cultured with Exo-MI. Compared with the transfection of inhibitor-NC, NRK-52E cells were transfected with the miR-1-3p inhibitor, the Bcl-2 expression was markedly inhibited, and the Bax expression was remarkably increased (Fig. 3E). The transfection of the miR-1-3p inhibitor also increased the proportion of TUNEL positive cells (Fig. 3F), and the apoptosis rate of NRK-52E cells (Fig. 3G). In addition, the silencing miR-1-3p increased the ratio of LC3II/LC3I (Fig. 3H) reduced autophagosomes (Fig. 3I).

### The overexpression of miR-1-3p inhibited the CM-induced apoptosis and autophagy of NRK-52E cells

**Figure 4:**
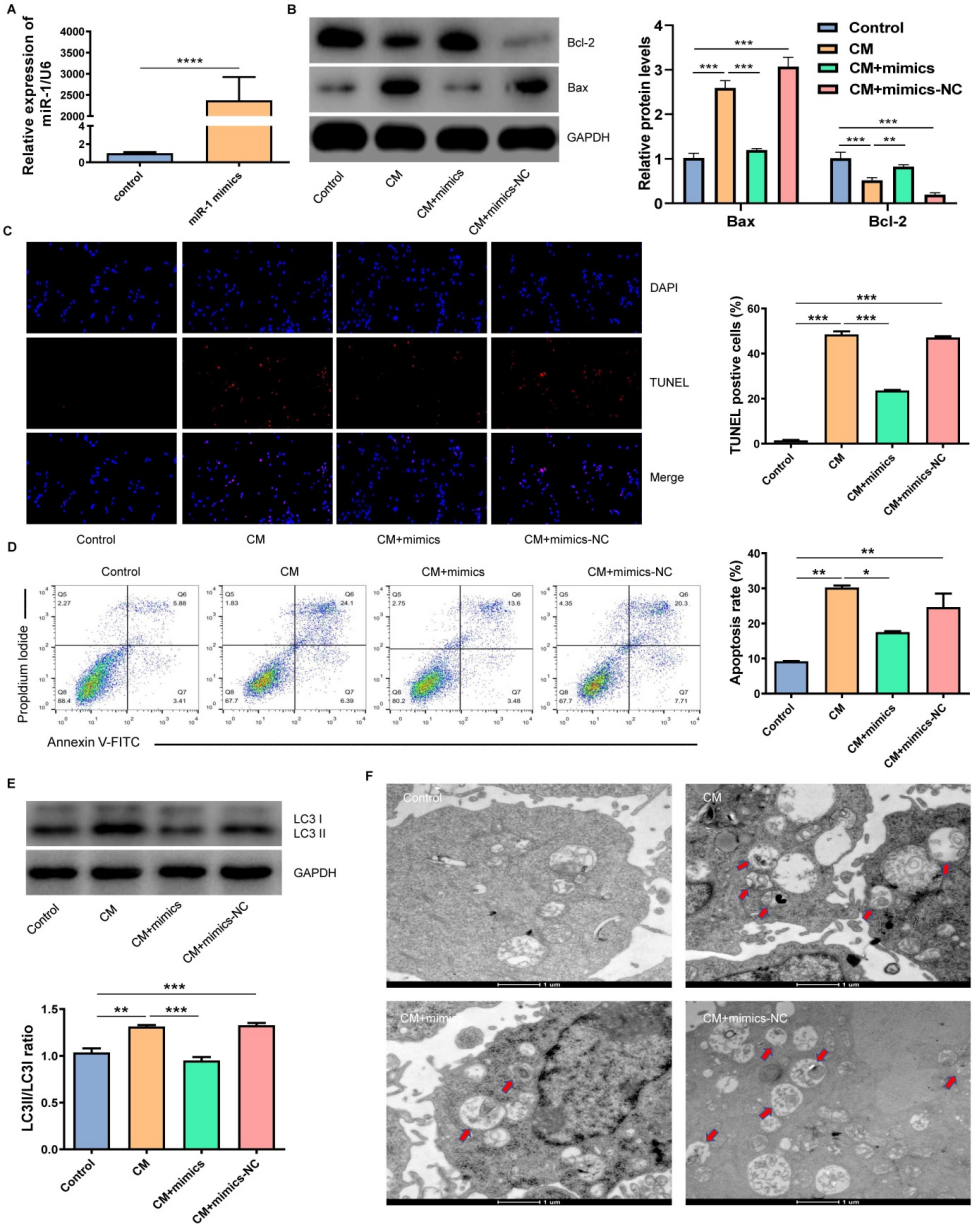
The overexpression of miR-1-3p inhibited the CM-induced apoptosis and autophagy of NRK-52E cells. (A) The expression of miR-1-3p in NRK-52E cells transfected with miR-1-3p mimics or mimics-NC was detected using RT-qPCR. (B) The expression of Bcl-2 and Bax in NRK-52E cells transfected with miR-1-3p mimics or mimics-NC was detected by WB. (C) The apoptosis of NRK-52E cells was detected by TUNEL staining (red). Magnification, ×200. (D) The apoptosis rate of NRK-52E cells was detected by FC. (E) The expression of autophagy-related proteins LC3I and LC3II in NRK-52E cells was detected by WB. (F) The morphology of autophagosomes was observed by TEM. The red arrow indicates autophagosomes. Scale bar, 1 μm. The data were expressed as mean ± standard error of mean (n=3). *P<0.05, **P<0.01, ***P<0.001.

In order to verify whether miR-1-3p played a key role in the protection of NRK-52E cells from CM injury, we transfected miR-1-3p mimics into NRK-52E cells to up-regulate the miR-1-3p expression (Fig. 4A). Figure 4B showed that the overexpression of miR-1-3p significantly reduced Bax expression and increased Bcl-2 expression in NRK-52E cells induced by CM. The overexpression of miR-1-3p could also reduce the number of CM-induced TUNEL-positive cells (Fig. 4C). Similarly, FC showed that the upregulation of miR-1-3p could also greatly reduce the apoptotic percentage of NRK-52E cells induced by CM (Fig. 4D). The ratio of LC3II/LC3I in the CM + mimics group was significantly lower than that in the CM group (Fig. 4E). The number of autophagosomes in the CM + mimics group reduced markedly compared with the CM + mimics-NC group (Fig. 4F). These results indicate that the overexpression of miR-1-3p can significantly inhibit the CM-induced apoptosis and autophagy of NRK-52E cells.

### MiR-1-3p directly targets ATG13 and activates the AKT signaling pathway

**Figure 5:**
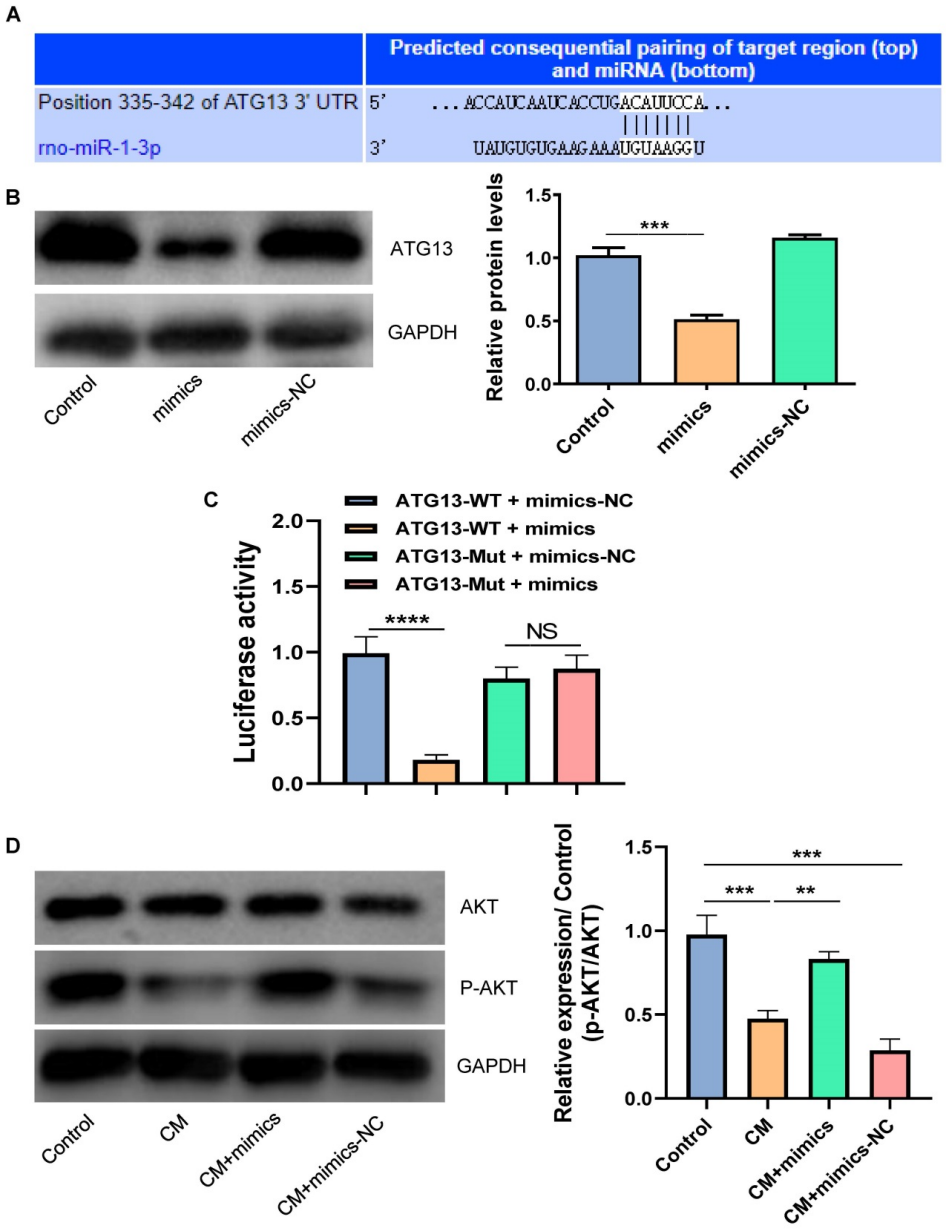
The miR-1-3p directly targets ATG13 and activates the AKT signaling pathway. (A) The target site of miR-1-3p binding to ATG13 mRNA predicted by the TargetScan prediction tool. (B) The expression of ATG13 in NRK-52E cells transfected with miR-1-3p mimics or mimics-NC was detected by WB. (C) The dual luciferase reporter assay has proven that miR-1-3p directly targets ATG13 mRNA. (D) The expression of AKT and P-AKT in NRK-52E cells transfected with miR-1-3p mimics or mimics-NC was detected by WB. The data were expressed as mean ± standard error of mean (n=3). **P<0.01, ***P<0.001, ****P<0.0001; NS, represents no statistical significance.

By using the TargetScan tool, we found that ATG13 might be a target gene of miR-1-3p (Fig. 5A). We further disclosed the regulatory relationship between miR-1-3p and ATG13 by WB. Figure 5B showed that the overexpression of miR-1-3p could markedly reduce the expression of ATG13. Most importantly, the luciferase activity assay demonstrated that the overexpression of miR-1-3p could visibly inhibit the luciferase activity in the wild-type group, but not in the mutant group (Fig. 5C). Furthermore, the WB revealed that the phosphorylation level of AKT was markedly decreased in NRK-52E cells after exposure with CM. However, after miR-1-3p was up-regulated, the phosphorylation level of AKT visibly increased (Fig. 5D). As a result, miR-1-3p directly targets ATG13, and the overexpression of miR-1-3p can activate the AKT signaling pathway.

### ATG13 silencing inhibited the CM-induced apoptosis and autophagy of NRK-52E cells

**Figure 6:**
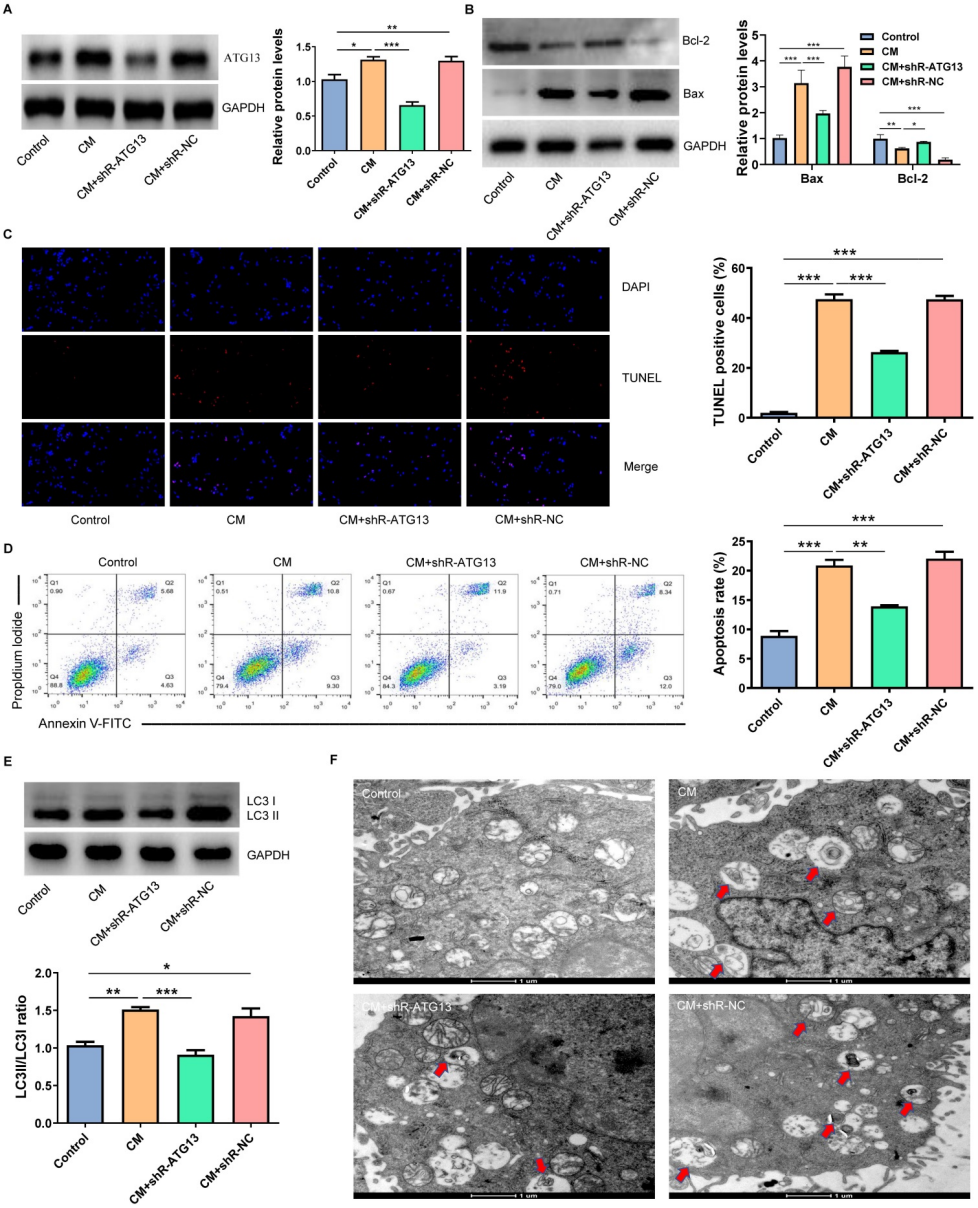
The ATG13 silencing inhibited the CM-induced apoptosis and autophagy of NRK-52E cells. (A) The expression of ATG13 in NRK-52E cells transfected with plasmids containing shR-ATG13 or shR-NC was detected by WB. (B) The expression of Bcl-2 and Bax in NRK-52E cells transfected with plasmids containing shR-ATG13 or shR-NC was detected by WB. (C) The apoptosis of NRK-52E cells transfected with plasmids containing shR-ATG13 or shR-NC was detected by TUNEL staining (red). Magnification, ×200. (D) The apoptosis rate of NRK-52E cells transfected with plasmids containing shR-ATG13 or shR-NC was detected by FC. (E) The expression of LC3I and LC3II in NRK-52E cells transfected with plasmids containing shR-ATG13 or shR-NC was detected by WB. (F) The morphology of autophagosomes was observed by TEM. The red arrow indicates the autophagosomes. Scale bar, 1 μm. The data were expressed as mean ± standard error of mean (n=3). *P<0.05, **P<0.01, ***P<0.001.

In order to investigate the role of ATG13 in CIN, plasmids that contain shR-ATG13 or shR-NC were transfected into NRK-52E cells. The WB revealed that the expression of ATG13 in the CM group significantly increased compared with the control group. Furthermore, shR-ATG13 significantly inhibited the protein expression of ATG13 (Fig. 6A). Then, we also examined the apoptosis and autophagy of NRK-52E cells after silencing ATG13. The silencing of ATG13 inhibited the CM-induced apoptosis of NRK-52E cells with promoting the expression of Bcl-2, inhibiting the expression of Bax, and reducing the number of TUNEL positive cells (Figs. 6B-6D). In addition, the ATG13 silencing inhibited the CM-induced autophagy in NRK-52E cells with reducing the ratio of LC3II/LC3I, and the number of autophagosomes decreased (Figs. 6E and 6F). These results indicate that the ATG13 silencing can inhibit the CM-induced apoptosis and autophagy of NRK-52E cells.

### Exo-MI alleviated CIN and improved the kidney function of rats

**Figure 7:**
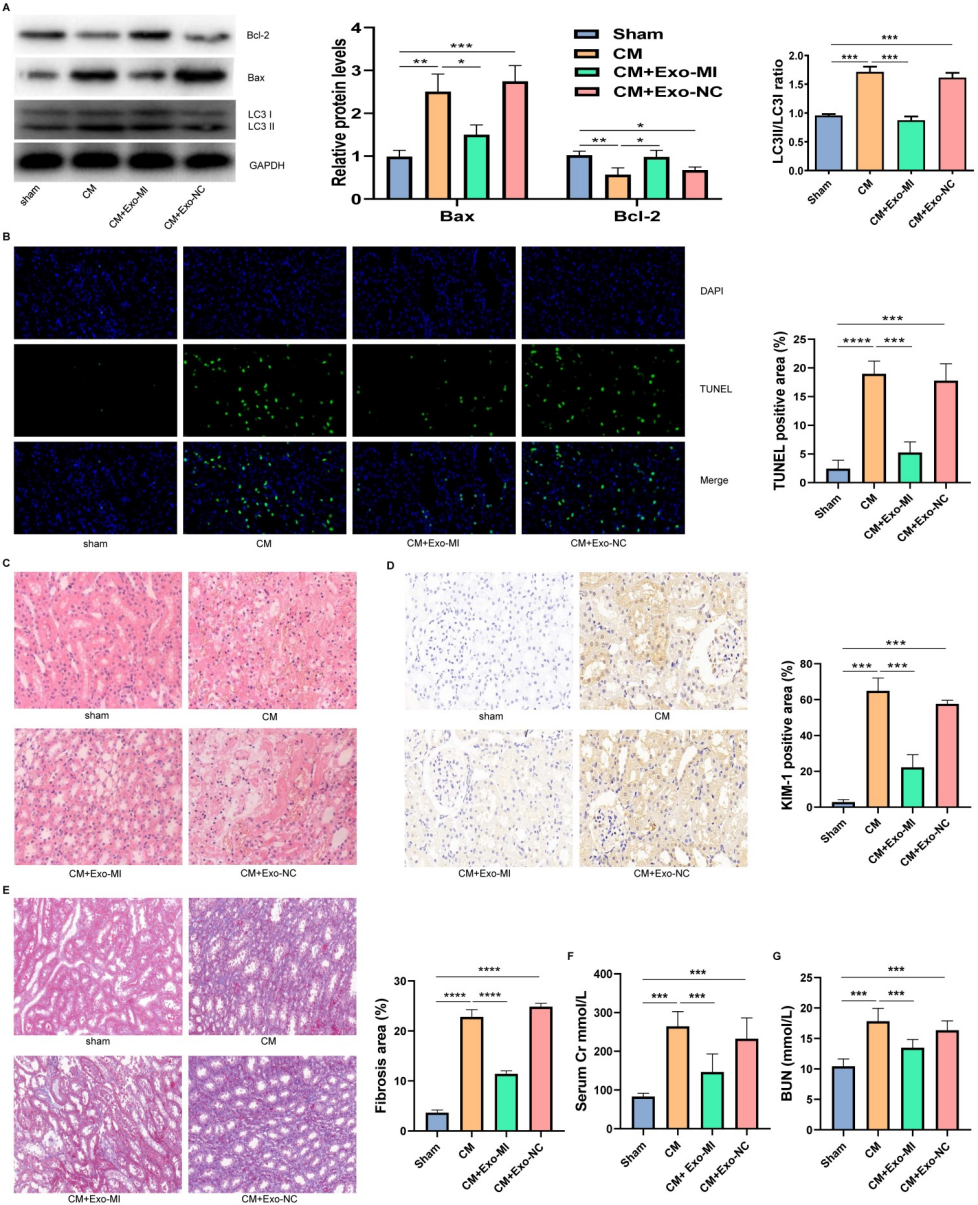
The Exo-MI alleviated the CIN and improved the kidney function of rats. (A) The levels of Bcl-2, Bax, LC3I and LC3II in kidney tissues were detected by WB. (B) Apoptosis of renal tissues was detected by TUNEL staining (red). Magnification: ×400. (C) The pathological changes of renal tissues of rats were evaluated by H&E staining. Magnification: ×400. (D) The renal tubular epithelial cell damage was assessed by KIM-1 immunohistochemical staining. Magnification: ×400. (E) The renal fibrosis was assessed by Masson's trichrome staining. Magnification: ×200. (F and G) The levels of BUN and serum Cr were tested to assess the renal function of rats. The data were expressed as mean ± standard error of the mean (n=3). *P<0.05, **P<0.01, ***P<0.001, ****P<0.0001.

We extracted the Exo-MI and injected these into CIN rats via the tail vein. The CM obviously induced apoptosis and autophagy in the kidney, promoted the expression of Bax, inhibited the expression of Bcl-2, and increased the ratio of LC3II/LC3I. However, the Exo-MI could significantly reverse these effects (Fig. 7A). The TUNEL staining of kidney tissues revealed that the Exo-MI could significantly inhibit the apoptosis of kidney cells induced by CM (Fig. 7B). The H&E staining revealed that the cytoplasm of the kidney tissue in the control group was evenly stained, and the cell morphology was generally normal, while the CM group and CM+Exo-NC group presented with the obvious destruction of the renal tissue structure, renal tubular edema, and cell necrosis, while the kidney damage in the CM + Exo-MI group was significantly alleviated (Fig. 7C). Kidney injury molecule-1 (KIM-1) has a significantly enhanced expression after renal tubular epithelial cell injury, and is an indicator that can quickly and sensitively reflect the process of kidney injury. The KIM-1 immunohistochemical staining revealed that CM can obviously induce the expression of KIM-1, which can be suppressed by Exo-MI (Fig. 7D). In addition, we also assessed the fibrosis level of kidney tissue by Masson's trichrome staining. Compared with the control group, the renal fibrosis was obvious in the CM group and CM + Exo-NC group, while Exo-MI significantly inhibited the CM-induced renal fibrosis (Fig. 7E). Furthermore, we tested the levels of BUN and serum Cr to assess the rat kidney function. The Exo-MI significantly reversed the CM induced increase BUN and serum Cr levels (Figs. 7F and 7G). These results indicate that Exo-MI can alleviate the CM-induced renal injury and improve the renal function of CIN rats.

## Discussion

In the present study, we have identified a previously unknown mechanism through which ischemic myocardium sends "signals" to the kidney. The main findings are as follows: Firstly, the expression of miR-1-3p significantly increased in the infarcted myocardium and circulating exosomes. Secondly, the kidney can take up exosomes secreted by cardiomyocytes. Thirdly, the circulating exosomal miR-1-3p from MI rats can protect renal function from CM-induced renal injury. Fourthly, the protective effect of miR-1-3p on CIN was largely achieved by targeting ATG13 via AKT signaling pathway.

The role of stem cell-derived exosomes in cardiac repair after MI has been extensively studied 13-15, but the physiological role of circulating exosomes secreted by the myocardium after MI is still unclear. As an important tool for cell-to-cell communication, exosomes are involved in the regulation of physiological and pathological processes in various organs 9, 16. Exosomes exist stably in body fluids, and can realize information interaction between different cells through circulation. This increases the possibility that miRNAs be detected in the circulation, and play a therapeutic role in target organs. Cheng et al 17 reported that bone marrow mononuclear cells could absorb circulating exosomes from MI mice and participate in the repair of myocardium. Our study revealed that exosomes secreted by ischemic myocardium can diminish CM-induced apoptosis and autophagy of renal tubular epithelial cells, which is an important mechanism of CIN. These findings indicated that the heart can communicate with distant organs through exosomes, thereby achieving a variety of biological functions, which is worthy of further exploration.

Studies have reported that after MI, the content of miRNAs in exosomes secreted by myocardium will change, including miR-1. When MI occurs, the expression of miR-1 in circulation is significantly increased 18, 19. And myocardial specific miR-1 is primarily carried by exosomes in the circulation 17. A previous report confirmed that exosomes mediate transfer of miR-1 into kidney 20. Our study also confirmed *in vitro* that renal tubular epithelial cells can absorb circulating exosomal miR-1 and overexpression of miR-1 could protect kidney against CIN via regulating the apoptosis and autophagy of kidney.

Apoptosis and autophagy play an important role in the development of CIN. In an *in vitro* study, the presence of apoptosis was confirmed by nuclear fragmentation and activation of Caspase-3 and Caspase-9 after the use of CM 21. Romano et al 22 reported that the use of CM reduced Bcl-2 expression and increased BAX, BAD, and BCL-2-like protein 11 (BCL2L11) expression in renal cells. These results are consistent with ours. However, the role of autophagy in the pathogenesis of CIN is controversial. Some researchers believe that autophagy can cause renal injury, but some scholars have suggested that autophagy is a protective factor in the development of CIN. Buyuklu et al 23 reported that CM can promote the expression of LC3II and cleaved Caspase-3 in kidney tissue, and after the treatment was given, the expressions of LC3II and cleaved Caspase-3 were reduced, and the renal function of rats was improved. However, Gang et al 24 found that CM can promote LC3 expression in renal tubular epithelial cells, but the use of autophagy inhibitors can induce the apoptosis of tubular epithelial cells and cause further decrease in the renal function of mice. In this study, it was also confirmed that the use of CM induces autophagy in the kidney, but the difference is that we found that inhibiting autophagy can reduce kidney injury. We speculate that this may be partly due to the different kinds and dosages of CM. As a form of cell death, the effect of autophagy on cells is not constant. Mild autophagy maintains cellular vitality by degrading impaired organelles to produce adenosine triphosphate (ATP), but excessive autophagy promotes cell death 25-27. Therefore, the opposite effect of inhibiting autophagy in CIN seems to be understandable.

ATG13 not only plays an important role in autophagy, but also participates in the regulation of apoptosis. The activation of the autophagy pathway depends on the ULK complex, which is an important node in the cellular autophagy network, and the ATG13 protein is an important subunit of the ULK complex 28, 29. And ATG13 can interact with Fas-associating protein with a novel death domain (FADD) to promote cell apoptosis 30. Through dual luciferase reporter assays, ATG13 was validated to be the target of miR-1. In addition, overexpression of miR-1 or ATG13 silencing can inhibit the apoptosis and autophagy of renal tubular epithelial cells induced by CM. Therefore, miR-1 and ATG13 may become potential targets for the treatment of CIN.

There are some limitations in this present study. First, we did not study other myocardial specific miRNAs, such as miR-208 and miR-499, which can also be absorbed by the kidneys through exosomes 17. Second, the ideal CIN model *in vivo* should be a CIN model of MI rats. However, due to the high mortality of rats after 2 operations, we chose to extract exosomes from MI rats and inject them into CIN rats through the tail vein. Third, the role of AKT signaling pathway in CIN was not studied in depth.

## Conclusion

In summary, it was found that the circulating exosomal miR-1-3p expression significantly increased in MI rats, and was absorbed by the kidneys through blood circulation, resulting in increased levels of miR-1-3p in renal tubular epithelial cells. Furthermore, miR-1-3p directly targets ATG13 and then activates the AKT signaling pathway, thereby inhibiting the CM-induced apoptosis and autophagy of renal tubular epithelial cells, and improving the renal function of rats.

## Figures and Tables

**Table 1:** Primer sequences of related genes for reverse-transcription quantitative polymerase chain reaction

Gene name	Forward (5'>3')	Reverse (5'>3')
miR-1-3p cel-miR-39 U6	AGGCGTGGAATGTAAAGAAGT GGGTCACCGGGTGTAAATC CTCGCTTCGGCAGCACA	GCGTTGTGTTGTGTTGTGTT GAGAGGAGAGGAAGAGGGAA AACGCTTCACGAATTTGCGT

RT-qPCR, reverse-transcription quantitative polymerase chain reaction

## Abbreviations

MI: myocardial infarction
AMI: acute myocardial infarction
CIN: contrast-induced nephropathy
CM: contrast medium
Exo-NC: circulating exosomes from control rats
Exo-MI: circulating exosomes from MI rat
RT-qPCR: Reverse transcription quantitative polymerase chain reaction
TUNEL: terminal deoxynucleotidyl transferase (TdT)-mediated dUTP nick end labeling
NTA: nanoparticle tracking analysis
TEM: transmission electron microscope
H&E: hematoxylin and eosin
KIM-1: kidney injury molecule-1
PCI: percutaneous coronary intervention
miRNA: microRNA
lnRNA: long noncoding RNA
DMEM/F12: Dulbecco's Modified Eagle's Medium/F12
FBS: fetal bovine serum
shR-ATG13: plasmids containing ATG13 small hairpin RNA
shR-NC: negative control small hairpin RNA
mimics-NC: miR-1-3p mimics negative control
PBS: phosphate buffered saline
ATG13: autophagy related protein 13
BUN: blood urea nitrogen
Cr: creatinine
PI: propidium iodide
CCK-8: cell counting kit-8
FADD: Fas-associating protein with a novel death domain
ATP: adenosine triphosphate
