# Circulating Exosomal miR-1-3p from Rats with Myocardial Infarction Plays a Protective Effect on Contrast-Induced Nephropathy via Targeting ATG13 and activating the AKT Signaling Pathway: Erratum

**DOI:** 10.7150/ijbs.86961

**Published:** 2023-10-21

**Authors:** Pengcheng Zhao, Yeqian Zhu, Ling Sun, Wenwu Zhu, Yao Lu, Jian Zhang, Yangming Mao, Qiushi Chen, Fengxiang Zhang

**Affiliations:** 1 Section of Pacing and Electrophysiology, Division of Cardiology, the First Affiliated Hospital with Nanjing Medical University, Nanjing, China.; 2 Department of Cardiology, the Affiliated Changzhou No. 2 People's Hospital of Nanjing Medical University, Changzhou, China.

In our paper, Fig. 2 should be corrected because an error was introduced in typesetting the figure. We found that there were overlaps between CM group and CM+Exo-NC group in Fig 2F. After careful comparison of the original data, we found that the two figures with overlapping parts in the paper were both from the CM group, because that we mistakenly placed the CM group pictures in the CM+Exo-NC group when typesetting the pictures. We declare that the correction does not change the results or conclusions of this paper. In this regard, all authors have agreed to the erratum, and we sincerely apologize for having this error in the article, and apologize for any inconvenience caused.

Figure 2F should be corrected as follows.

## Figures and Tables

**Figure 2:**
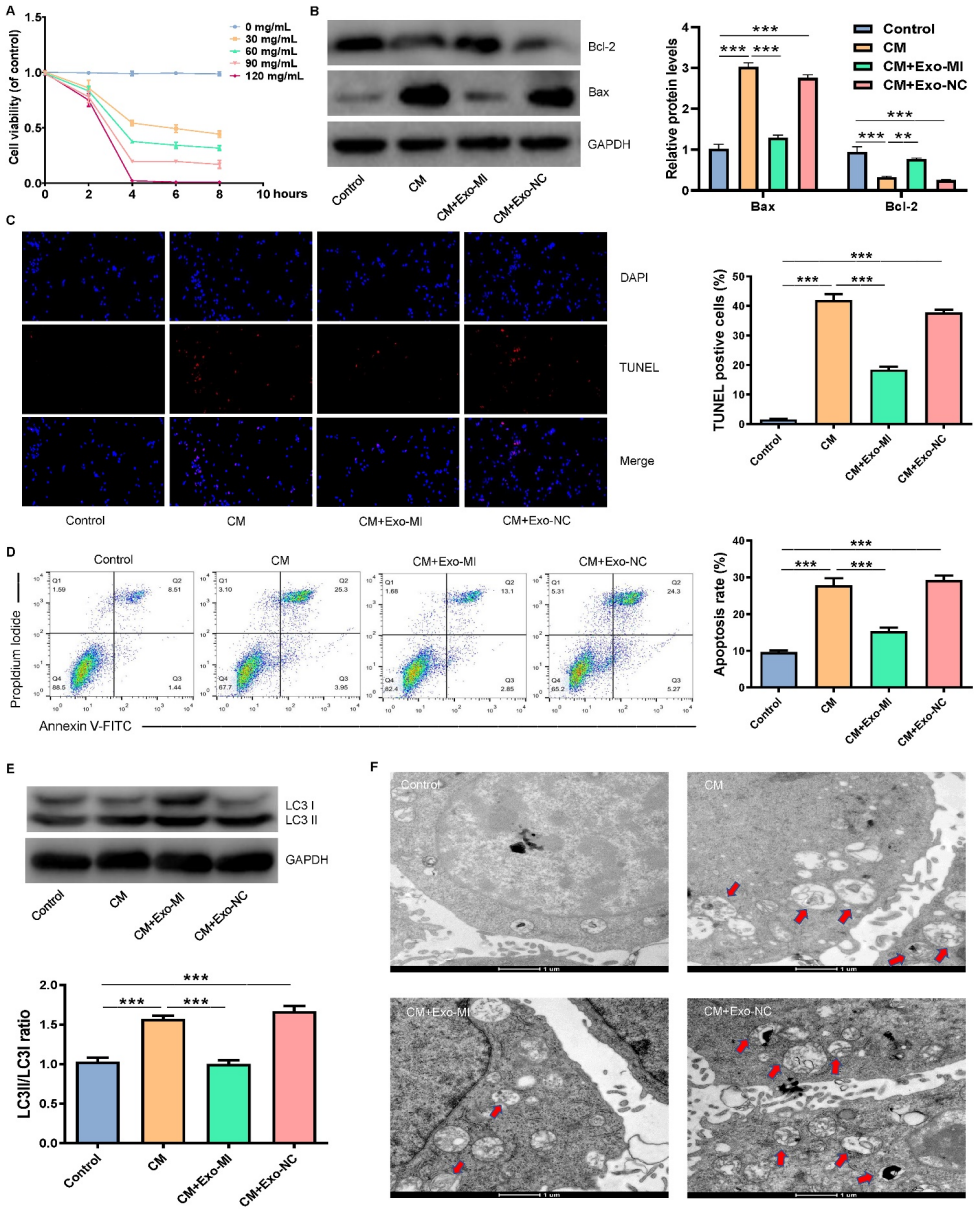
Correct image.

